# A case of delayed-onset postoperative pyoderma gangrenosum complicated with chronic myelomonocytic leukemia treated with systemic corticosteroids and spesolimab

**DOI:** 10.1016/j.jdcr.2025.11.053

**Published:** 2025-12-17

**Authors:** Gong Zhenni, Tao Siqi, He Li, Ma Xiaoya, Jia Xuesong, Ci Hai, Wang Xue

**Affiliations:** a Department of Dermatology, The First Affiliated Hospital of Shihezi University, Shihezi, China; b Shihezi University School of Medicine, Shihezi, China; c Department of Burn and Plastic Surgery, The First Affiliated Hospital of Shihezi University, Shihezi, China

**Keywords:** chronic myelomonocytic leukemia, delayed-onset, postoperative pyoderma gangrenosum, spesolimab

## Introduction

Pyoderma gangrenosum (PG) constitutes an uncommon form of neutrophilic dermatosis frequently linked to systemic diseases, notably hematologic malignancies. Chronic myelomonocytic leukemia (CMML) provides a distinctive framework for the pathogenesis of PG, attributable to the mutual dysregulation of myeloid cell function. This case report documenting the delayed emergence of PG in conjunction with CMML postsurgery underscores the pivotal role of inhibiting the interleukin 36 (IL-36) pathway in managing PG associated with CMML.

## Case report

A 63-year-old male patient underwent a laparoscopic tension-free repair of a unilateral inguinal hernia. Medical history includes hypertension, cerebral infarction, and a status poststenting for coronary atherosclerotic disease. On the 15th postoperative day, he presented to the clinic with persistent pain at the umbilical incision site, where a 2 × 1 cm^2^ ulcer had formed. The surgeon observed the presence of white necrotic tissue and purulent discharge at the base of the ulcer, indicating a potential cutaneous infection. The obtained cultures were negative. Consequently, the decision was made to commence empirical treatment with ceftriaxone for ongoing concern for infection. However, after a duration of 5 days, the ulcerated region had further enlarged, and the pain had increased in severity. Therefore, the administration of ceftriaxone was terminated. On the 20th postoperative day, the patient was admitted for further medical intervention owing to alterations in the surgical incision. The wound area enlarged to approximately 10 × 6 cm^2^, with the wound margins presenting raised, purplish-blue skin.

**Fig 1:**
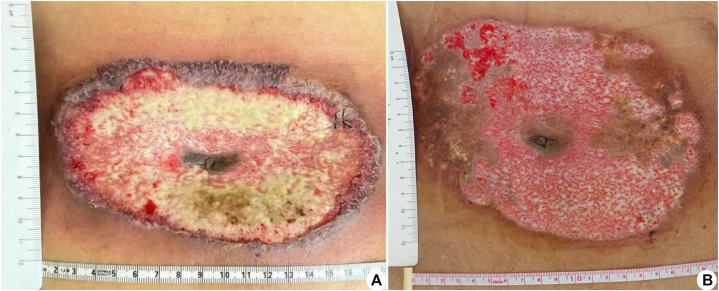
Clinical photograph of the wound (umbilical surgical site): **(A)** There is a 9 × 18 cm^2^ necrotic ulcer, with violaceous undermined borders and a peripheral inflammatory halo. Characteristic “tissue-paper” necrosis can be seen at the edge. **B,** The ulcer was significantly reduced to 8 × 5 cm^2^, and the central area was covered with homogenous pink granular tissue, with a moist surface and a small amount of plasma exudation; the margins showed advancing epithelialization in the form of a reddish rolled edge. The original satellite pustular lesion is absorbed, leaving a punctuate atrophic scarring, which is painless on palpation.

**Fig 2:**
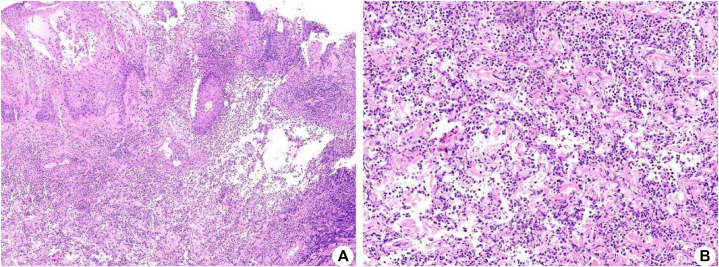
Hematoxylin and Eosin of PG. **A,** There was local ulcer formation, the dermis and subcutaneous tissue showed obvious hyperplasia of deep fibers and inflammatory granulation tissue, accompanied by a large number of acute and chronic inflammatory cell infitration as well as abscess formation (Hematoxylin and Eosin staining ×40); **(B)** A large mumber of acute and chronic inflammatory cells, including neutrophils and multinuclear giant cells, were noted in the dermis (Hematoxylin and Eosin staining ×100). *PG*, Pyoderma gangrenosum.

On the second day of hospitalization, the patient developed a fever with a peak temperature of 38 °C and was started on ceftazidime to address the infection. Despite a 3-day course of ceftazidime, the fever recurred. The wound expanded further to 18 × 9 cm^2^, presenting a 1 cm-wide margin of elevated blackish-purple ischemic skin tissue (Fig 1, *A*). Scattered tension blisters were observed. As a result, antibiotic therapy was intensified to piperacillin-tazobactam. During this period, the wound underwent multiple debridements and dressing changes. Repeat wound and blood cultures remained negative, and the patient's wound area continued to expand. Considering the presence of skin necrosis and potential diagnosis of PG, a skin biopsy was deemed necessary. A skin biopsy demonstrated diffuse neutrophil infiltration within the dermal layer (Fig 2). The diagnosis of PG was made on the basis of the characteristic clinical presentation and supportive histopathological findings. Following this, the administration of antibiotics was discontinued. We administered an intravenous infusion of 80 mg (roughly 1 mg/kg dosing) of methylprednisolone. This steroid dose was continued for 7 days and then decreased over 5 weeks. The patient's ulcer demonstrated a progressive reduction in size, which was accompanied by a decrease in exudation and the emergence of skin islands. Fever and pain resolved.

Laboratory findings showed anemia and leukocytosis. Normal results included liver/renal function, tumor markers, autoantibodies, and vasculitis panels. A bone marrow biopsy, augmented by immunohistochemistry, identified a myeloid tumor, characterized by approximately 5% of primitive naive cells and graded as myelofibrosis-0. The utilization of flow cytometric immunofluorescence indicated that approximately 1.24% of myeloid primitive cells presented immunophenotypic abnormalities. Genetic testing revealed mutations in ASXL1, CSF3R, SETBP1, and STAG2. Final diagnoses were PG and CMML. In the third week of methylprednisolone use, the patient received a single intravenous dose of 900 mg of spesolimab (Table I). The ulcerated area exhibited substantial reduction, measuring 8 × 5 cm^2^, and the central region displayed uniform pink granular tissue. The surface remained moist with minimal serous exudate, while the margins exhibited raised, reddened epithelialization (Fig 1, *B*).

**Table I:** Timeline of therapy

Date	Time point	Therapeutic intervention	Dosage/regimen	Ulcer status
April 16	15 d postsurgery	IV ceftriaxone	2 g/d	2 × 1 cm^2^ ulcer with pale necrotic tissue and purulent discharge is visible at the base
April 22	Admission day (D 0)	IV ceftazidime	2 g/q12 h	10 × 6 cm^2^ ulcer with pale necrotic tissue and discharge. Raised, purplish-blue skin around wound margins
April 25	D 4	IV piperacillin-tazobactam	4.5 g/q8 h	18 × 9 cm^2^ ulcer with a 1 cm wide violaceous undermined borders and a peripheral inflammatory halo.
April 28	D 7	IV methylprednisolone	80 mg/d	20 × 10 cm^2^ ulcer and the wound surface has not changed
May 5	D 14	IV methylprednisolone	70 mg/d	18 × 13 cm^2^ ulcer with visible skin island formation and significantly reduced exudate
May 19	D 28	IV spesolimab	900 mg	8 × 5 cm^2^ ulcer, with the central region exhibiting uniform coverage by granular pink tissue

At the 2-month follow-up, the patient demonstrated a notable reduction in pain level. The wound dimensions had diminished to 6 × 3 cm^2^ and exhibited considerable healing. Nonetheless, conspicuous red granular tissue persisted in localized regions, accompanied by observations of hyperpigmentation and hypopigmentation at the wound peripheries.

## Discussion

PG is an infrequent neutrophilic dermatosis characterized histologically by a pronounced infiltration of neutrophils and heightened expression of cytokines, including IL-36.^1^ CMML, identified as a clonal hematopoietic stem cell disorder, is typified by persistent monocytosis and irregular cytokine signaling pathways.^2^ The pathophysiology of CMML involves an aberration in myeloid cell function, which results in the augmented secretion of proinflammatory factors such as IL-1β, IL-6, TNF-α, and IL-36.^3^ These factors not only propagate inflammation within the bone marrow microenvironment but may also elicit immune responses in remote tissues, such as the skin, via the circulatory system. Common epigenetic mutations associated with CMML (eg, ASXL1, TET2, and PHF6) potentially aggravate systemic inflammation by impairing the function of immune cells.^4^

Activation of the IL-36 receptor (IL-36R) triggers the NF-κB and MAPK signaling cascades, leading to the release of chemokines such as IL-8 and CXCL1 from keratinocytes and neutrophils. This process recruits additional neutrophils to the dermal region, thereby perpetuating a vicious cycle of inflammation.^5^ The presence of CMML alongside PG suggests that aberrant monocytes originating from CMML may persistently secrete cytokines such as IL-36, thereby activating the neutrophilic inflammatory response within the skin and potentially inducing or aggravating PG skin lesions.^6^

From a therapeutic standpoint, conventional immunosuppressants are frequently associated with insufficient efficacy and elevated recurrence rates in patients with PG who also present with hematologic malignancies. Spesolimab, a highly selective IL-36R monoclonal antibody, effectively inhibits IL-36 signaling to suppress neutrophil chemotaxis and activation, thereby disrupting the central inflammatory pathway of PG.^7^ In this investigation, the patient experienced expedited ulcer healing subsequent to combined steroid and spesolimab treatment, substantiating the therapeutic merit of targeting the IL-36 pathway. The drug has received approval for the treatment of generalized pustular psoriasis, with its safety affirmed in clinical trials.^8^

Moreover, the diagnosis of postoperative PG presents significant challenges and is frequently mistaken for a surgical site infection.^9^ In this instance, the persistent progression of skin lesions despite repeated debridements is consistent with the paradoxical clinical presentation characteristic of postoperative pyoderma gangrenosum.

## Conflicts of interest

None disclosed.
